# The Working Girl

**Published:** 1890-06

**Authors:** 


### THE WORKING GIRL.

We meant to say a word in commendation of the Working Girl's Congress recently held in New York city, before this.

A more able, orderly, and well mannered assembly has scarcely ever convened in our midst. Their three days' session was distinguished for candor, ability and good sense. The grievances of the working girl, her long hours of toil and half-paid earnings, were discussed and commented upon in a way to enlist the sympathy of all classes, save their well housed and over fed employers.

There would seem to be a natural remedy for this great and growing evil, in the establishment of industrial co-operative societies and stores for the sale of their handiwork, but attempts in this direction have for the most part resulted in failure, with no fault on the part of their projectors. The difficulty is, that those of their own sex in circumstances of affluence, withhold their patronage, preferring to pay some well-to-do shopkeeper a higher price for a less valuable article, the profits of which are ground out of midnight hours and starvation wages. It is a sorrowful truth that as between the woman of fashion and the working girl, there is little or no sympathy. This is notably the case in instances of sudden and unexpected opulence.

It is only the noble and self assured that rises so far above conditions, as to leave them wholly out of mind in the endeavor to benefit her toiling sisters, who seek not charity but justice.

